# High-throughput drug screening identifies the ATR-CHK1 pathway as a therapeutic vulnerability of *CALR* mutated hematopoietic cells

**DOI:** 10.1038/s41408-021-00531-2

**Published:** 2021-07-31

**Authors:** Ruochen Jia, Leon Kutzner, Anna Koren, Kathrin Runggatscher, Peter Májek, André C. Müller, Michael Schuster, Christoph Bock, Joanna I. Loizou, Stefan Kubicek, Robert Kralovics

**Affiliations:** 1 Department of Laboratory Medicine, Medical University of Vienna, Vienna, Austria; 2 CeMM Research Center for Molecular Medicine of the Austrian Academy of Sciences, Vienna, Austria; 3 Institute of Artificial Intelligence, Center for Medical Statistics, Informatics, and Intelligent Systems, Medical University of Vienna, Vienna, Austria; 4 Department of Medicine I, Comprehensive Cancer Center, Medical University of Vienna, Vienna, Austria

**Keywords:** Preclinical research, Drug development

## Abstract

Mutations of calreticulin (CALR) are the second most prevalent driver mutations in essential thrombocythemia and primary myelofibrosis. To identify potential targeted therapies for *CALR* mutated myeloproliferative neoplasms, we searched for small molecules that selectively inhibit the growth of *CALR* mutated cells using high-throughput drug screening. We investigated 89 172 compounds using isogenic cell lines carrying *CALR* mutations and identified synthetic lethality with compounds targeting the ATR-CHK1 pathway. The selective inhibitory effect of these compounds was validated in a co-culture assay of *CALR* mutated and wild-type cells. Of the tested compounds, CHK1 inhibitors potently depleted *CALR* mutated cells, allowing wild-type cell dominance in the co-culture over time. Neither *CALR* deficient cells nor *JAK2V617F* mutated cells showed hypersensitivity to ATR-CHK1 inhibition, thus suggesting specificity for the oncogenic activation by the mutant CALR. CHK1 inhibitors induced replication stress in *CALR* mutated cells revealed by elevated pan-nuclear staining for γH2AX and hyperphosphorylation of RPA2. This was accompanied by S-phase cell cycle arrest due to incomplete DNA replication. Transcriptomic and phosphoproteomic analyses revealed a replication stress signature caused by oncogenic CALR, suggesting an intrinsic vulnerability to CHK1 perturbation. This study reveals the ATR-CHK1 pathway as a potential therapeutic target in *CALR* mutated hematopoietic cells.

## Introduction

Classical myeloproliferative neoplasms (MPNs) are a group of clonal hematopoietic diseases that are driven by somatic mutations acquired by hematopoietic stem/progenitor cells. Three phenotypic driver mutations have been uncovered in MPNs including mutations in *JAK2*, *MPL*, and *CALR* genes, which collectively are genetic determinants of more than 95% of MPN cases [1–8]. The discovery of these oncogenes led to the emergence of novel targeted therapies for MPNs. Notably, JAK2 inhibitors were developed a few years after the discovery of the *JAK2V617F* mutation and achieved great clinical success [9–11].

Mutations of *CALR* are the most common driver mutations in *JAK2(V617F)*-negative ET and PMF patients. All *CALR* mutations are frameshift mutations that occur in exon 9 of the gene, all utilizing the same alternative reading frame [7, 8]. The mutant calreticulin activates pro-proliferative JAK-STAT signaling through the thrombopoietin receptor (MPL), leading to uncontrolled proliferation and clonal expansion of the *CALR* mutated cells [12–17]. Patients with mutations in *CALR* often harbor a high mutant allele burden, suggesting *CALR* mutations to be strong clonal drivers [18]. Recent research revealed additional molecular features of cells with mutations in *CALR*, such as an elevated unfolded protein response and abnormalities in calcium signaling [19, 20]. However, none of these features have been exploited sufficiently to develop novel drug treatments. In a recent study, our group demonstrated the possibility of targeting the glycan-binding pocket of mutant CALR protein by small molecules as a chemotherapeutic approach [21].

To search for new drug targets and to identify clinical compounds for the treatment of *CALR* mutated MPN, we performed an unbiased high-throughput screen of a large compound library in *CALR* mutated isogenic cell lines. This led to the identification of several checkpoint kinase 1 (CHK1) and ataxia telangiectasia and Rad3-related protein (ATR) inhibitors as promising candidates that selectively inhibited the growth of *CALR* mutated cell lines. Since replication stress has been linked to several oncogenes, resulting in the vulnerability of the malignant cells to ATR-CHK1 inhibitors [22–25], we further validated these compounds and revealed a replication stress signature in the *CALR* mutated cells that is causative for the synthetic lethal interaction.

## Materials and methods

### Cell lines

Endogenous CALR mutations were generated using CRISPR-Cas9 in UT-7/Tpo and Ba/F3-MPL cell lines. The mutated cell lines were characterized and cultured as previously described [16, 21]. In both cases, gRNAs were designed to cut at the site where deletion 52 bp mutation starts. No DNA template of a specific mutation type was provided. The sequence information of the CALR mutated clones was provided in Supplementary figure 1.

### High-throughput drug screen

The screen was performed in collaboration with Platform Austria for Chemical Biology (PLACEBO) using a customized library of 89172 compounds. UT-7/Tpo cells were seeded at a density of 16,000 cells/well in 384-well plates. UT-7/Tpo CALR del61/WT cell line was used for the initial cytotoxicity screen. Both UT-7/Tpo parental and UT-7/Tpo CALR del61/WT cell lines were used for the follow-up selectivity screen. Each condition had 3 technical replicates. CellTiter-Glo assay was used to measure cell viability after 72 h of incubation. The proteasomal inhibitor Bortezomib was used as the positive control at an assay concentration of 10 µM in the primary screen and 13.5 µM in the secondary screen. All compounds were added as DMSO solution, and an equivalent amount of DMSO (0.1% in the primary screen, 0.135% in the secondary screen) was used as the negative control. The Z-factor was calculated for each plate [26]. Only the plates with a Z-factor > 0 passed quality control and were taken into analysis.

To compare compounds across plates with different signals, a percentage of control (POC) was calculated by linear regression, setting the mean signal of the DMSO wells to 100% and the mean signal of the positive control wells to 0% for each plate individually.

### Chemicals

All compounds used for the initial screen and dose-response validation were provided by PLACEBO. For other assays, the following drugs and suppliers were used: Rabusertib/LY2603618 (Cayman Chemical Company, USA); SCH900776/MK8776 (MedChemExpress, USA); Adavosertib/MK-1775 (MedChemExpress, USA).

### Co-culture competition assay

Two-color competition assays were conducted by co-culturing the CALR wild type and mutated cells in the presence of compounds and 1% TPO cytokine supplement over 5–12 days. The two competing cell lines can be distinguished by FACS, as one expresses mCherry. The establishment of the mCherry expressing cell lines was described previously [21]. Three biological replicates were performed for each experiment.

### Western blot

UT-7/Tpo *CALR* WT, *CALR* del61/WT cells, and primary CD34+ cells were treated with the indicated drugs for 24 h. Cells were then washed twice with PBS (Gibco, USA). The cell pellets were used for protein extraction and Western blot analysis, as previously described [21]. All the antibodies used in this assay were listed in Supplementary Table 1.

### Colony formation assay

CD34+ cells were isolated from peripheral blood mononuclear cells (PBMCs) of 3 healthy individuals and 3 CALR patients. 1000 cells were plated with 0.25uM MK8776 or DMSO in MethoCult H4434 classic media (Stemcell Technologies, USA) for 10 days. The isolation of PBMCs and CD34+ cells was performed as previously described [21]. The use of the patients’ materials was approved by the ethics committee in the Medical University of Vienna in accordance with the Declaration of Helsinki. The written informed consent from the patients were given.

### Immunofluorescence

For immunofluorescent imaging, compound-treated cells were plated on 10% (v/v) poly L-lysine (Sigma) coated slides for 1 h at 37 °C for immobilization. Cells were then washed twice with PBS and fixed in 4% paraformaldehyde (PFA) (Santa Cruz Biotechnology, USA) at room temperature for 15 min. For γ-H2AX staining, a pre-extraction step was performed by adding 0.2% (v/v) Triton-X (Sigma-Aldrich, Germany) in PBS on slides for 1 min on ice. Permeabilization was performed using 0.5% Triton-X in PBS for 5 min at room temperature. Cells were then washed in PBS with 0.1% (v/v) Tween20 (Sigma-Aldrich, Germany) three times for 5 min. 5% (m/v) bovine serum albumin (BSA) (Sigma-Aldrich, Germany) in PBS with 0.1% Tween20 (Sigma-Aldrich, Germany) was used for blocking for 1 h at room temperature. Primary antibodies were then diluted in blocking buffer for overnight staining at 4 °C. Cells were then washed in PBS Tween solution 3 times before secondary antibody staining for 1 h at room temperature. Cells were again washed 3 times before nuclei were stained with 0.1% (m/v) DAPI (Sigma-Aldrich, Germany) for 10 min at room temperature. Cells were then washed twice and ProLong™ Gold Antifade Mountant medium (ThermoFisher Scientific, USA) was applied. The slides were covered with a coverslip and dried for 48 h at room temperature before analyzed or stored at 4 °C. The antibodies used in the assay were listed in Supplementary Table 2.

The Zeiss LSM700 confocal microscope (Leica Microsystems, Germany) was used for image scanning. The 405 nm laser was used for DAPI signal detection and the 639 nm laser was used to detect the Alexa-fluor 647 signal. The 20X (0.8NA) Plan-Apochromat objective lens and a pinhole size of 1 airy unit was used for image capture. Images were analyzed using the ZEN black 2010 software. Images from 3 biological replicates were used for analysis. Quantification of positively stained cells was performed by CellProfiler.

### Cell cycle analysis

Cell cycle analysis was performed using 10^6^ cells fixed in 70% ice-cold ethanol for a minimum of 2 h. Cells were then washed with PBS and blocked in 5% BSA solution for 1 h. First, anti-γ-H2AX antibody (1:1000) (Merck Millipore, USA) was used for staining of 60 min at room temperature, followed by washing in PBS and staining with anti-mouse IgG (H+L) Alexa Fluor 647 (1:1000) (Invitrogen, USA) for 60 min at room temperature in the dark. DNA staining was done using 1 mg/ml DAPI solution for 20 min at room temperature in the dark. Samples were analyzed by BD LSRFortessa™ (BD Biosciences, USA).

### Sample preparation for RNA sequencing and Mass spectrometry analysis

For RNA sequencing, CRISPR-Cas9 generated *CALR* heterozygous and homozygous mutated clones, and wild-type control Ba/F3-MPL cells were used. Cells were washed with PBS and then cultured in fresh RPMI 1640 medium (Gibco, USA) for 4 h before sample collection. Two million cells for each sample were collected and washed twice with DPBS. The cells were then centrifuged and cell pellets were stored at −80 °C before RNA extraction, using the RNeasy Mini Kit as per the manufacturer’s instructions (Qiagen, USA). The performance of RNA sequencing and data analysis are documented in the supplementary method.

Ba/F3-MPL CALR wild type, and CALR del37/del37 cells were seeded at the same density and then cultured for 8 h in fresh RPMI 1640 medium before sample collection. 20 million cells for each sample were collected, washed with PBS, and then pelleted. This procedure was performed three times to acquire three biological replicates for each sample. Two biological replicates of the CALR wild-type sample and three biological replicates of the CALR del37/del37 sample were used for mass spectrometry experiment and analysis. The procedure of sample processing and data analysis of mass spectrometry are described in the supplementary method.

### Statistics

For all the quantification assays, at least three biological replicates were performed and analyzed for statistical analysis. Two-way analysis of variance followed by Tukey’s multiple comparisons test was used for statistical analysis in the co-culture competition assay and the quantification of γH2AX positive cells by immunofluorescence. Paired *t*-test was used for the statistical comparison in the colony formation assay. 3 biological clones of each genotype (WT, heterozygous, homozygous) were cultured at the same density and collected as biological replicates for the RNA sequencing experiment. Three independent sample collection experiments were performed to collect the samples for the mass spectrometry experiment. Transcriptomic and phosphoproteomic analyses are described in detail in the supplementary method.

## Results

### ATR and CHK1 inhibitors selectively target *CALR* mutated cells

**Fig. 1:**
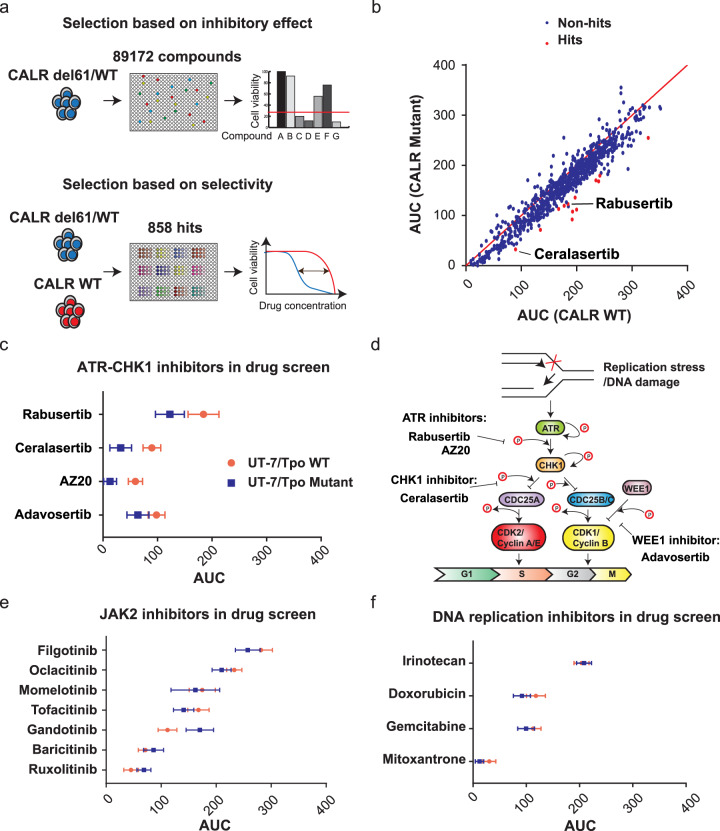
High-throughput compound screen identifies ATR-CHK1 inhibitors as synthetic lethal with mutant CALR. **a** A schematic diagram of compound screen workflow. The UT-7/Tpo *CALR* del61/WT cell line was used for the initial screen. Each compound was used at 10 µM or higher, in a 3-day cell viability assay. Selected compounds were used in the validation screen with both UT-7/Tpo *CALR WT* and UT-7-Tpo *CALR del61/WT* cell lines against 4 doses of each compound. **b** The plot of the area under the curve (AUC) of all the compounds tested in the validation screen. After the validation screen, the AUC was calculated as the sum of all percentage of control (POC) values of each drug in a 4-dose response. Each POC value was calculated as a mean of 3 technical replicates. Each dot represents one compound tested on the screen. Red dots indicate the compounds identified as selective inhibitors of *CALR* mutated cells. **c** A comparison of AUC of *CALR* WT and mutated cells from the four inhibitors of the ATR-CHK1 pathway. A larger AUC value indicates a lower inhibitory effect on the cell line. **d** A schematic diagram of the ATR-CHK1 pathway as well as the targets of the four ATR-CHK1 inhibitors. **e** A comparison of the AUC of *CALR* WT and mutated cells to the JAK2 inhibitors tested on the screen. **f** A comparison of the AUC of *CALR* WT and mutated cells to the DNA replication inhibitors tested on the screen.

To discover novel drug candidates for CALR mutated MPN, we screened a library of 89,172 compounds against UT-7/Tpo *CALR* wild type and mutated cell lines. To also exclude compounds with low biological activity, we used the library at a single-dose high concentration (typically 10 µM or higher) in a *CALR del61/WT* cell line (Fig. 1a). Next, we selected compounds that showed more than 75% of inhibitory effect on the tested cells for further investigation. In addition, those compounds that exhibited an inhibitory effect in the range of 50–75% but with known targets were also included for further validation. Altogether, 858 selected compounds were next tested in both UT-7-Tpo *CALR* wild type and mutated cell lines to generate 4-dose response curves. The selectivity of each compound was assessed by comparing its inhibitory effect in CALR wild type with mutated cell lines (Fig. 1a).

We calculated the area under the curve (AUC) value as a cumulative measure of compound potency. Compounds were selected as synthetic lethal with mutant CALR when the AUC of the mutant cell line was at least 50% lower than the wild-type cell line with at least one standard deviation difference between the two means. Using these criteria, we identified 13 compounds that showed selective inhibition in the *CALR* mutated cells. Interestingly, two out of thirteen inhibitors, ceralasertib, and rabusertib, target the ATR-CHK1 signaling pathway (Fig. 1b, c). This outcome prompted us to further examine additional ATR-CHK1 inhibitors included in the library. AZ20 (ATR inhibitor) and adavosertib (Wee1-like protein kinase inhibitor) also showed milder but selective inhibition in *CALR* mutated cells (Fig. 1c). Adavosertib inhibits the phosphorylation of cyclin-dependent kinase 1 (CDK1) by WEE1 kinase, which regulates the G2-M cell cycle checkpoint [27]. As multiple compounds targeting the ATR-CHK1 pathway were identified in this unbiased screen, we hypothesized that there is a specific vulnerability of CALR mutated cells to the inhibition of this pathway (Fig. 1d).

Since the CALR mutant oncoprotein together with MPL activates the JAK-STAT signaling pathway, we also investigated the performance of JAK2 inhibitors in the screen. *CALR* mutated cells did not show significant hypersensitivity to any of the tested JAK2 inhibitors, suggesting that these compounds do not specifically target *CALR* transformed cells (Fig. 1e).

The drug sensitivity of cell lines might vary due to different proliferation rates, as fast-growing cells might be more susceptible to drugs affecting DNA replication. Therefore, we also analyzed four genotoxic drugs inhibiting DNA replication or metabolism. None of these drugs showed preferential inhibition of *CALR* mutated cells, indicating that the global inhibition of DNA replication cannot explain the selective sensitivity to ATR-CHK1 inhibitors (Fig. 1f).

### CHK1 inhibitors are synthetic lethal with *CALR* mutated cells

**Fig. 2:**
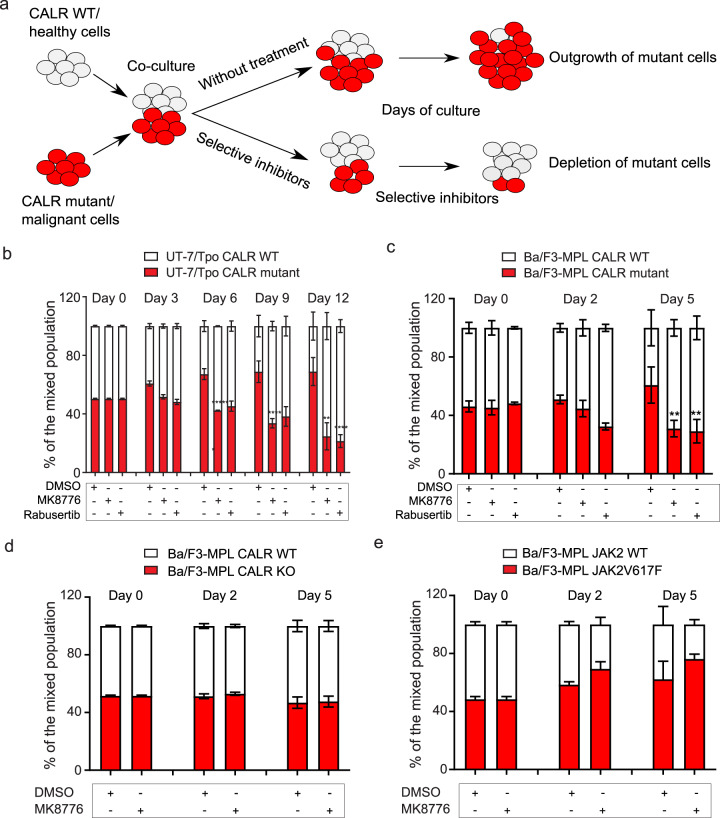
CHK1 inhibitors selectively reduce the proliferation of cells carrying oncogenic CALR. **a** Schematic diagram of the setup of the co-culture competition assay. **b** UT-7/Tpo *CALR* wild type and del61/WT cell line were incubated with 0.8 µM of CHK1 inhibitor rabusertib or 1 µM of MK8776 over 12 days. Wild type (mCherry negative) and mutant cells (mCherry positive) were mixed at a 1:1 ratio and 1% of TPO conditioned media was present. Samples were collected every three days and the ratio of wild-type cells to mutant cells was measured by FACs analysis. **c** Ba/F3-MPL *CALR* wild type and del37/del37 cell line were incubated with 0.8 µM rabusertib or 1 µM MK8776 over 5 days. Wild type (mCherry negative) and mutant cells (mCherry positive) were mixed at a 1:1 ratio and 1% of TPO conditioned media was present. Samples were collected at day 0, day 2, and the end of treatment. The ratio of wild-type cells to mutant cells was measured by FACs analysis. **d** Ba/F3-MPL *CALR* wild type and *CALR* knockout cell line were incubated with 1 µM MK8776 over 5 days in a co-culture competition assay. **e** Ba/F3-MPL *JAK2* wild type and *JAK2V617F* mutated cell lines were incubated with 1 µM MK8776 over 5 days in a co-culture competition assay.

We chose to focus on CHK1 inhibitors for further validation, as they showed more robust selectivity on the screen (Fig. 1c). We also included a further CHK1 inhibitor, MK8776, that was not included in the compound library. We set up a two-color competition assay by co-culturing *CALR* wild type and mutated cells in the presence of the tested inhibitors (Fig. 2a). By transducing either CALR mutant or control cell line with a construct carrying mCherry, we could distinguish the two populations and detect selective cytotoxicity of the drug over a longer time than a standard proliferation assay. As shown in Fig. 2b, UT-7/TPO *CALR* mutated cells outgrew wild-type cells in the presence of DMSO, whereas in the presence of CHK1 inhibitors rabusertib or MK8776, the clonal expansion of mutant cells was diminished over time (Fig. 2b). To rule out the influence of mCherry transduction on the fitness of the cells, we labeled wild-type cells with mCherry and confirmed the result (Supplementary figure 2).

To rule out a cell line-dependent effect, we repeated the experiment using a murine Ba/F3-MPL cell line. Compared to UT-7/Tpo, Ba/F3 cells proliferate more rapidly. Therefore, 5 days of treatment with CHK1 inhibitors resulted in a significant suppression in the growth of *CALR* mutated cells (Fig. 2c). We also showed that CHK1 inhibitor at the concentration used in this assay did not kill most of the wild-type cells (Supplementary figure 3).

To evaluate the effect of CHK1 inhibition on the primary hematopoietic cells from CALR mutated patients. We performed colony formation assay using CD34+ cells isolated from healthy donors and CALR patients and then applied MK8776 treatment. A statistically significant reduction of colonies following MK8776 treatment was detected for CALR patient samples (Supplementary figure 4).

To evaluate the specificity of this gene-drug interaction of mutant CALR, we generated *CALR* knockout Ba/F3-MPL cell lines and performed the competition assay co-cultured with wild-type control cells. In the presence of the CHK1 inhibitor MK8776, the ratio of the wild type to CALR deficient cells did not change (Fig. 2d). This finding confirms that CHK1 inhibition specifically reduces cell proliferation in the presence of the oncogenic CALR mutant.

Next, we tested the drug in cells carrying the JAK2V617F mutation and did not observe selective cytotoxicity, suggesting that the synthetic lethal interaction between CHK1 and CALR mutation is specific as opposed to a general outcome due to activation of the JAK-STAT pathway (Fig. 2e).

### CHK1 inhibition induces replication stress in *CALR* mutated cells

**Fig. 3:**
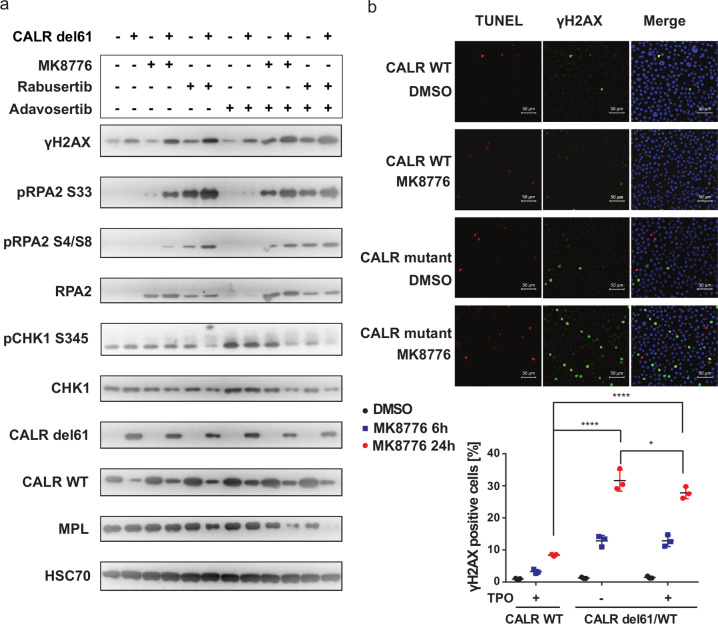
CHK1 inhibition induces elevated γ-H2AX and RPA phosphorylation in *CALR* mutated cells. **a** Western blot analysis of UT-7/Tpo *CALR* *WT* and *CALR del61/WT* whole cell lysates following treatment of the indicated drugs for 24 h. 1 µM MK8776, 0.8 µM rabusertib, and 0.3 µM adavosertib were used as single-agent treatment. 0.2 µM adavosertib combined with 0.17 µM MK8776 or 0.13 µM rabusertib were used for combination treatment. **b** Top panel: Immunofluorescence of UT-7/Tpo *CALR WT* and *CALR del61/WT* following 1 µM MK8776 treatment for 6 h. Pseudocolor annotation: red represents TUNEL; green represents γ-H2AX; blue represents DAPI. Bottom panel: Quantification of γ-H2AX positive cells in UT-7/Tpo *CALR WT* and *CALR del61/WT* cells after 6 and 24 h treatment of MK8776. *p < 0.05; ****p < 0.0001.

To explore the underlying mechanism that results in the selective inhibition of *CALR* mutant cells, we probed for DNA damage response markers in UT-7/Tpo cells in the presence of the drug treatment. CHK1 inhibitors rabusertib and MK8776 were used as single agents and in combination with WEE1 inhibitor adavosertib, as the synergistic effect was reported when combined CHK1 and WEE1 inhibitors [27, 28]. Even without treatment, we observed elevated γ-H2AX in *CALR* mutated cells compared to the wild-type cells, suggesting that oncogenic mutant CALR increased DNA damage response signaling above basal levels (Fig. 3a). In the presence of CHK1 inhibitors alone or in combination with adavosertib, a more pronounced induction of γ-H2AX was detected in the *CALR* mutated cells compared to the control, indicating that more DNA damage or replication stress was triggered in the *CALR* mutated cells (Fig. 3a). We also examined replication protein A (RPA), the single-strand DNA binding protein that stabilizes replication forks. Hyperphosphorylation of its subunit RPA2 is a marker of the cellular response to DNA replication stress [29]. *CALR* mutated cells showed stronger phosphorylation at both S33 and S4/S8 sites upon CHK1 inhibitor treatment compared to control, which confirmed stronger replication stress was induced in the mutant cells. We used the phosphorylation at S345 of CHK1 as a marker of CHK1 inhibition, as it should be induced upon treatment with CHK1 inhibitors [30]. Interestingly, when compared to the two CHK1 inhibitors, rabusertib showed a weaker effect than MK8776 on CHK1 phosphorylation but a stronger effect on inducing γ-H2AX and RPA phosphorylation in both wild type and mutant cells. This suggests stronger general toxicity of rabusertib compared to MK8776 in wild-type cells. In comparison with CHK1 inhibitors alone, the combination of CHK1 and WEE1 inhibitors showed a stronger induction of H2AX and RPA2 phosphorylation, which is consistent with previous reports (Fig. 3a).

To strengthen the evidence for increased signaling of replication stress in CALR mutant cells upon CHK1 inhibition, we imaged γ-H2AX in UT-7 cells following MK8776 treatment for 6 and 24 h. An increase in γ-H2AX was observed, in CALR mutant cells exposed to CHK1 inhibitor treatment (Fig. 3b top and bottom panels). We excluded the possibility that apoptotic cells with fragmented DNA were leading to the increased γ-H2AX signal by co-staining with TUNEL (terminal deoxynucleotidyl transferase dUTP nick end labeling).

We also performed a Western blot and detected an upregulation of γ-H2AX induced by MK8776 in CD34+ cells derived from a CALR patient, whereas γ-H2AX remained at the basal level for the healthy control with the same treatment (Supplementary figure 5).

**Fig. 4:**
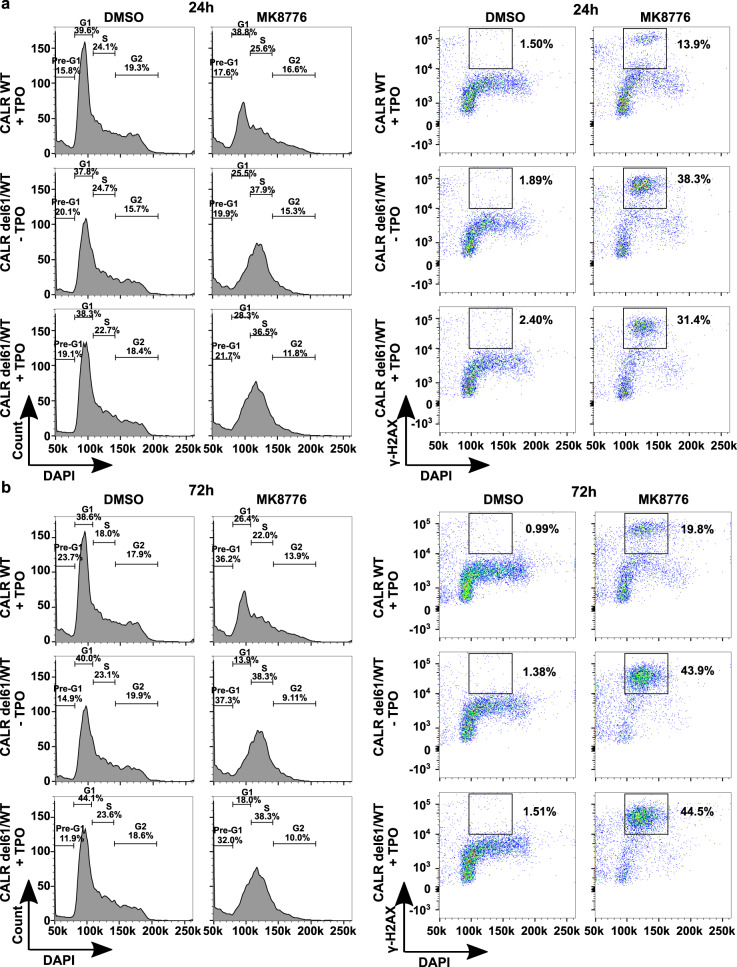
CHK1 inhibitors induce S-phase cell cycle arrest in *CALR* mutated cells. **a** Left panel: histogram of UT-7/Tpo *CALR WT* and *CALR del61/WT* cell lines treated with 1 µM MK8776 or DMSO for 24 h, followed by DAPI staining to indicate DNA content. The percentage of cells at pre-G1, G1, S, G2-M phases is labeled in the graphs. Right panel: FACS co-staining of DAPI and γ-H2AX at a 24 h time point post-treatment. γ-H2AX positive S-phase cells are labeled and the percentage of this population is indicated in the graphs. **b** Left panel: histogram of UT-7/Tpo *CALR WT* and *CALR del61/WT* cell lines treated with 1 µM MK8776 or DMSO for 72 h, followed by DAPI staining to indicate DNA content. The percentage of cells at pre-G1, G1, S, G2-M phases is labeled in the graphs. Right panel: FACS co-staining of DAPI and γ-H2AX at a 72 h time point post-treatment. γ-H2AX positive S-phase cells are labeled and the percentage of this population is indicated in the graphs.

Since replication stress is associated with cell cycle arrest, we analyzed the cell cycle distribution upon CHK1 inhibition. After 24 h treatment with MK8776, an S-phase cell cycle arrest was induced in the mutant cells, in the presence or absence of TPO (Fig. 4a left panel). When DAPI was co-stained with γ-H2AX, nearly all the S-phase *CALR* mutated cells were γ-H2AX positive when treated with MK8776, compared to a significantly lower percentage (3-fold difference) of γ-H2AX positive cells in the control cell line (Fig. 4a right panel). We also observed a lower fraction of mutant cells in G2 upon treatment. Continuation of the treatment to 72 h caused most of the mutant cells to become blocked in S-phase and these cells were γ-H2AX positive (Fig. 4b). Taken together, the data support the conclusion that CALR mutated cells accumulate replication stress and S-phase arrest upon CHK1 inhibition.

### Expression of oncogenic CALR leads to a replication stress signature revealed through alterations in the transcriptome and phosphoproteome

**Fig. 5:**
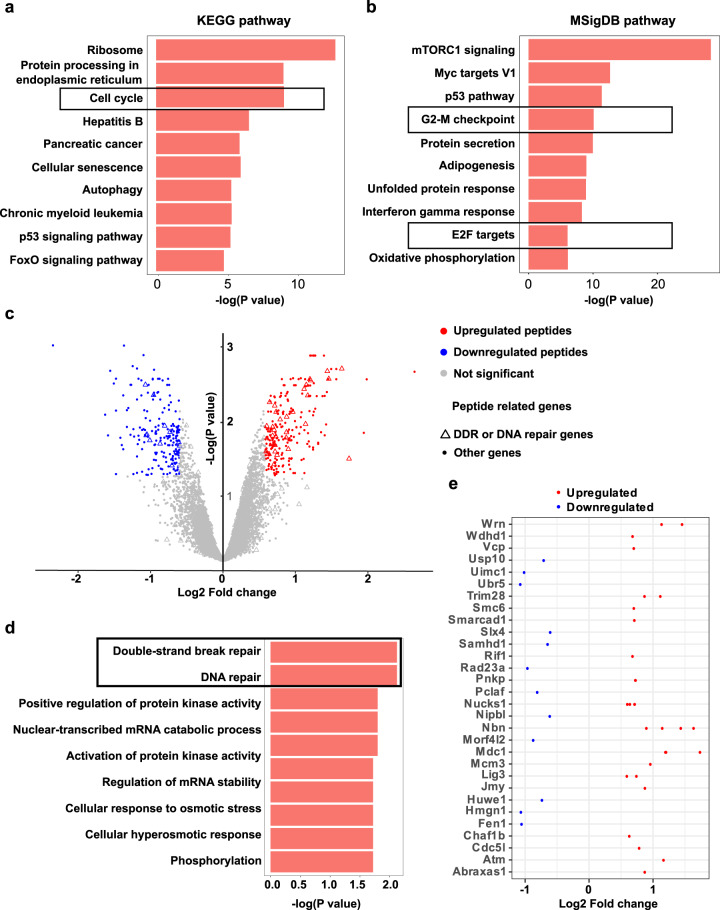
Transcriptomic and phosphoproteomic analyses reveals a replication stress signature caused by the oncogenic CALR. **a** RNA-sequencing identified differentially expressed genes in Ba/F3-MPL CALR mutated cells compared to CALR WT cells. The gene-set enrichment analysis was performed by Enrichr. The top10 most enriched KEGG pathways are shown. **b** The top 10 most enriched MSigDB pathways are shown in the gene-set enrichment analysis. **c** Volcano plot of differentially phosphorylated peptides identified by phosphoproteomic analysis. Each dot represents a phosphorylated peptide. A statistical significance threshold was set as at least a 1.5-fold change of abundance difference between the compared groups with a *p*-value lower than 0.05. The red and blue dots indicate upregulated and downregulated peptides, respectively, in the *CALR* mutant group. **d** The top 10 most enriched gene ontology biological processes are shown in the gene-set enrichment analysis of the differentially phosphorylated peptides. **e** Depiction of the 42 differentially phosphorylated peptides related to DNA damage response and repair pathways. Each dot represents a peptide. When multiple peptides of one gene are shown, it indicates the protein has more than one phosphorylation site that was identified. Upregulated/Downregulated indicates up or downregulation of the peptides in the *CALR* mutant group compared to the wild-type group.

As replication stress and activation of the DNA damage response can broadly impact the expression of genes involved in cell cycle checkpoints, we hypothesized that expression of the *CALR* oncogene may trigger this dysregulation upon CHK1 inhibition. To better understand the molecular consequences of oncogenic CALR expression, we analyzed changes in gene expression using an RNA sequencing experiment (RNA-seq) in Ba/F3-MPL cells expressing mutant and wild-type *CALR* (Fig. 5a, b). The gene-set enrichment analysis revealed significantly altered expression of cell cycle-related genes in *CALR* mutated cells, demonstrated by the pathway terms “Cell cycle”, “G2-M checkpoint” and “E2F targets” being among the most significantly enriched pathways (Fig. 5a, b, supplementary figure 6) [31–33].

Because phosphorylation is an important post-translational modification that regulates the DNA damage response, we analyzed changes in the phospho-proteome by mass spectrometry. Among the 431 differentially expressed phosphopeptides, we detected an overrepresentation (42/431) of DNA repair-related peptides, with the majority being upregulated in *CALR* mutated cells (Fig. 5c). A gene ontology analysis revealed “Double-strand break repair” and “DNA repair” to be the only biological processes that were highly significantly altered in the mutant cells (*P*-value < 0.01) (Fig. 5d). Upon closer investigation, we identified upregulation of phospho-ATM at Ser1981, phosphorylation of the Mre11-Rad50-Nbs1 (MRN) complex as well as MDC1 in the mutant cells (Fig. 5e), indicative of an activation of the DNA damage response. To summarize, both transcriptomic and phosphoproteomic data support our conclusions that the expression of mutant CALR impacts DNA replication and the DNA damage response, providing an explanation for the vulnerability to CHK1 inhibition.

## Discussion

Patients diagnosed with MPNs, notably myelofibrosis, require novel therapeutic approaches that can eliminate the hematopoietic clones carrying the molecular drivers. Mutant calreticulin has been identified as the driver of 25–35% ET and PMF patients [8], making it an attractive drug target for therapy. Surprisingly, although high-throughput drug screening has gained popularity as a powerful tool in biomedical research, no such effort has been made in the search for drugs targeting *CALR* mutated cells. In this study, we utilized a high content compound screen using a comprehensive, chemically diverse library consisting of many drugs at clinical stages for different indications as well as novel chemical compounds (Fig. 1).

Although we used a stringent threshold for the prioritization of selected compounds (less than 0.015%), two of the most significant compounds identified were found to target the ATR-CHK1 pathway. In contrast, neither JAK2 inhibitors nor genotoxic drugs showed the preferential killing of *CALR* mutated cells. Drugs targeting the DNA damage response and repair usually require several replication cycles to accumulate DNA damage leading to cytotoxicity. Therefore, we validated the most significant compounds in a co-culture competition assay over an extended period and also using a cell line with a shorter doubling time (Fig. 2c). These results revealed a synthetic lethal interaction between mutant *CALR* and CHK1 inhibitors (Fig. 2b).

We first hypothesized that the vulnerability of *CALR* mutated cells to the perturbation of DNA damage response and DNA repair could be due to a loss of wild-type calreticulin function, as two studies have identified enrichment of calreticulin at the stalled replication fork [34, 35]. We excluded this possibility since *CALR* knockout was not hypersensitive to CHK1 inhibition (Fig. 2d). Therefore, we argued that the hypersensitivity of *CALR* mutated cells is driven by the expression of an oncogene. Several studies have linked MPN with DNA damage and repair pathways. An elevation of reactive oxygen species and DNA double-strand breaks were reported in a *JAK2V617F* mutated model [36]. Also, impairment of replication fork progression and a defective cell cycle checkpoint was observed in *JAK2V617F* patient samples [37]. A more recent study showed that MPN driver mutations upregulate components of homologous recombination and non-homologous end-joining pathways to cope with increased DNA double-strand breaks [19]. However, no direct link between the DNA damage response and CALR mutations has been reported thus far. Since the ATR-CHK1 pathway plays a central role in the cellular response to stalled replication forks caused by replication stress, we hypothesized that elevated levels of replication stress might be caused by mutant CALR-driven proliferation. To investigate the presence of replication stress in *CALR* mutated cells, we investigated cellular markers of replication stress upon the CHK1 inhibition (Fig. 3a, b). RPA is a direct downstream target of ATR [38]. The hyperphosphorylation of its subunit, RPA2, is an important cellular response to replication stress, leading to apoptosis of cells upon CHK1 inhibition [29]. We observed marked upregulation of phosphorylated RPA at two phosphorylation sites in *CALR* mutated cells compared to the wild-type control when treated with CHK1 inhibitors and in combination with a WEE1 inhibitor (Fig. 3a). We also detected an elevation of γH2AX in *CALR* mutated cells, both at baseline and upon CHK1 inhibition (Fig. 3a). γH2AX is a marker of both DNA double-strand breaks and replication stress, but the pan-nuclear phosphorylation of H2AX is a unique marker of replication stress-induced cell death [39, 40]. We observed a significantly higher percentage of cells with pan-nuclear γH2AX in *CALR* mutated cells upon CHK1 inhibition, which supports our conclusion that replication stress is the cause of CHK1 inhibition-induced cell death when mutant CALR is present (Fig. 3b).

As CHK1 inhibition might cause replication catastrophe, leading to the breakage of replication forks and cell death [41], we performed cell cycle analysis and co-stained the cells with γH2AX. We observed an altered cell cycle distribution of the *CALR* mutated cells upon CHK1 inhibition, with a significantly higher fraction of the cells in the S-phase and unable to progress to the G2 phase (Fig. 4a). These cells were stained positive with γH2AX, suggesting the presence of collapsed replication forks (Fig. 4b).

As the hypersensitivity of the *CALR* mutated cells to CHK1 inhibition might suggest an intrinsic vulnerability to replication stress induction, we analyzed both gene expression and signaling alterations of untreated *CALR* mutated and wild-type cells. Differential gene expression analysis identified an enrichment of cell cycle-related genes when the oncogenic CALR is present (Fig. 5a, b), which is consistent to the single-cell RNA sequencing data of the hematopoietic stem and progenitor cells from CALR mutated patients [42]. Moreover, approximately 10% of the differentially phosphorylated peptides were related to DNA damage response and repair, suggesting abnormalities in this pathway as a main feature of the *CALR* mutated cells (Fig. 5c, d).

Many oncogenes, such as c-Myc, H-RAS, AKT, and CDC25A have been linked to the induction of replication stress and genomic instability through different mechanisms [24, 25, 43]. Our data suggest that mutated *CALR* shares this feature with other oncogenes. Interestingly, the *JAK2V617F* oncogene does not have this property (Fig. 2d). Although *JAK2*, *CALR*, and *MPL* mutations all induce the JAK-STAT pathway as the main oncogenic signaling, the cells carrying these mutations might still respond differently to CHK1 inhibition, due to the different oncogenic mechanisms. For instance, although CALR mutants are mainly present as heterozygous mutations, CALR mutated patients tend to have a higher mutant allele burden than the JAK2 mutated patients [18]. This suggests that CALR mutations might be stronger clonal drivers than the JAK2 mutation, which correlates with the cell proliferation and replication stress of the mutated cells. Also, the strong activation of MAPK signaling in CALR mutated cells was reported [44], which suggests that the signaling profile of the CALR and MPL mutations are not identical, despite both mutations induce their oncogenic signaling through the MPL receptor. Our study suggests that the inhibition of the ATR-CHK1 pathway might be therapeutically exploited in CALR mutated MPNs. Multiple CHK1 inhibitors and ATR inhibitors are at different stages of clinical development in several malignancies [45–47]. Exploring such inhibitors in *CALR* mutated MPNs would significantly shorten the time required to make a new therapeutic modality available to MPN patients who currently have a limited repertoire of treatment options.

## Supplementary information

Supplementary materials

## Data Availability

The mass spectrometry proteomics data have been deposited to the ProteomeXchange Consortium via the PRIDE [48] partner repository with the dataset identifier PXD026803 and 10.6019/PXD026803. The RNA sequencing data will be provided upon request.
