# Supplementary material for: High-throughput drug screening identifies the ATR-CHK1 pathway as a therapeutic vulnerability of CALR mutated hematopoietic cells
Source: Blood Cancer J. 2021 Jul 31;11(7):137. doi: 10.1038/s41408-021-00531-2 (PMC8325683; doi:10.1038/s41408-021-00531-2)
Supplement: Supplementary file 1 — Supplementary materials [file 41408_2021_531_MOESM1_ESM.docx]

**Supplementary method**

**NGS Library Preparation**

The amount of total RNA was quantified using the Qubit 2.0 Fluorometric Quantitation system (Thermo Fisher Scientific, Waltham, MA, USA) and the RNA integrity number (RIN) was determined using the Experion Automated Electrophoresis System (Bio-Rad, Hercules, CA, USA). RNA-seq libraries were prepared with the TruSeq Stranded mRNA LT sample preparation kit (Illumina, San Diego, CA, USA) using Sciclone and Zephyr liquid handling workstations (PerkinElmer, Waltham, MA, USA) for pre- and post-PCR steps, respectively. Library concentrations were quantified with the Qubit 2.0 Fluorometric Quantitation system (Life Technologies, Carlsbad, CA, USA) and the size distribution was assessed using the Experion Automated Electrophoresis System (Bio-Rad, Hercules, CA, USA). For sequencing, samples were diluted and pooled into NGS libraries in equimolar amounts.

**Next-Generation Sequencing and Raw Data Acquisition**

Expression profiling libraries were sequenced on HiSeq 3000/4000 instruments (Illumina, San Diego, CA, USA) following a 50-base-pair, single-end recipe. Raw data acquisition (HiSeq Control Software, HCS, HD 3.4.0.38) and base calling (Real-Time Analysis Software, RTA, 2.7.7) was performed on-instrument, while the subsequent raw data processing off the instruments involved two custom programs based on Picard tools (2.19.2) (1). In a first step, base calls were converted into lane-specific, multiplexed, unaligned BAM files suitable for long-term archival (IlluminaBasecallsToMultiplexSam, 2.19.2-CeMM). In a second step, archive BAM files were demultiplexed into sample-specific, unaligned BAM files (IlluminaSamDemux, 2.19.2-CeMM).

**Transcriptome Analysis**

NGS reads were mapped to the Genome Reference Consortium GRCm38 assembly via “Spliced Transcripts Alignment to a Reference” (STAR) (2) utilising the “basic” Ensembl transcript annotation from version e87 (December 2016) as reference transcriptome. Since the mm10 assembly flavour of the UCSC Genome Browser was preferred for downstream data processing with Bioconductor packages for entirely technical reasons, Ensembl transcript annotation had to be adjusted to UCSC Genome Browser sequence region names. STAR was run with options recommended by the ENCODE project. Aligned NGS reads overlapping Ensembl transcript features were counted with the Bioconductor (3.6) GenomicAlignments (1.14.0) package via the summarizeOverlaps function in Union mode, taking into account that the Illumina TruSeq stranded mRNA protocol leads to sequencing of the second strand so that all reads needed inverting before counting. Transcript-level counts were aggregated to gene-level counts and the Bioconductor DESeq2 (1.18.0) package (3) was used to test for differential expression based on a model using the negative binomial distribution.

An initial exploratory analysis included principal component analysis (PCA), multi-dimensional scaling (MDS), sample distance and expression heatmap plots, all annotated with variables used in the expression modelling (ggplot2, 2.2.1, and Bioconductor ComplexHeatmap, 1.15.1) (4,5). Biologically meaningful results were extracted from the model, two-tailed p-values obtained from Wald testing were adjusted with the Benjamini & Hochberg procedure. The resulting gene lists were annotated, filtered for significantly differentially up- and down-regulated genes and independently subjected to gene set enrichment analysis (Enrichr) (6–8).

**Sample preparation and phosphopeptide enrichment**

Each washed cell pellet was lysed separately in 40 μL of freshly prepared lysis buffer containing 50 mM HEPES (pH 8.0), 2% SDS, 0.1 M DTT, 1 mM PMSF, phosSTOP and protease inhibitor cocktail (Sigma-Aldrich). Samples rested at RT for 20 minutes before heating to 99 °C for 5 min. After cooling down to RT, DNA was sheared by sonication using a Covaris S2 high performance ultrasonicator. Cell debris was removed by centrifugation at 20.000 × g for 15 min at 20 °C. Supernatent was transferred to fresh eppendorf tubes and protein concentration determined using the BCA protein assay kit (Pierce Biotechnology, Rockford, IL). FASP was performed using a 30 kDa molecular weight cutoff filter (VIVACON 500; Sartorius Stedim Biotech GmbH, 37070 Goettingen, Germany) essentially according to the procedure described by Wisniewski *et al* (9). In brief, 200 µg total protein per sample were reduced by adding DTT at a final concentration of 83.3 mM followed by incubation at 99°C for 5 min. After cooling to room temperature, samples were mixed with 200 μL of freshly prepared 8 M urea in 100 mM Tris-HCl (pH 8.5) (UA-solution) in the filter unit and centrifuged at 14.000 × g for 15 min at 20 °C to remove SDS. Any residual SDS was washed out by a second washing step with 200 μL of UA. The proteins were alkylated with 100 μL of 50 mM iodoacetamide in the dark for 30 min at RT. Afterward, three washing steps with 100 μL of UA solution were performed, followed by three washing steps with 100µL of 50 mM TEAB buffer (Sigma-Aldrich). Proteins were digested with trypsin at a ratio of 1:50 overnight at 37 °C. Peptides were recovered using 40 μL of 50 mM TEAB buffer followed by 50 μL of 0.5 M NaCl (Sigma-Aldrich). Peptides were desalted using C18 solid phase extraction spin columns (The Nest Group, Southborough, MA). After desalting, 200 µg peptides were labeled with 0.8 mg TMT 10plex™ reagents at a 1:4 ratio (Pierce, Rockford, IL). After quenching of the labeling reaction, labeled peptides were pooled, organic solvent removed in vacuum concentrator and labelled peptides cleaned via C18 solid phase extraction (SPE). Peptides were eluted with 80% acetonitrile containing 0.1% trifluoroacetic at a final peptide concentration of ~1µg/µl. Eluate was then used for phosphopeptide enrichment applying a modified method of immobilized metal affinity chromatography (IMAC) published by (Ficarro et al., 2009). Briefly, 5 times 100 µL of Ni-NTA superflow slurry (QIAGEN Inc., Valencia, USA) were washed with LCMS-grade water and Ni^2+^ stripped off the beads by incubation with 100 mM of EDTA, pH 8 solution for 1 hr at room temperature. Stripped NTA resin was recharged with Fe^3+^-ions by incubation with a fresh solution of Fe(III)Cl_3_ and 100 µL of charged resin slurry used for the enrichment of a total of ~400 µg TMT-labelled peptides (in total 500µg Fe-IMAC for 2mg TMT-labeled peptide). The unbound fraction was transferred to a fresh glass vial and used for offline fractionation for the analysis of the whole proteome. After washing the slurry with 0.1% TFA, phosphopeptides were eluted with a freshly prepared ammonia solution containing 3mM EDTA, pH 8 and all used for offline fractionation for the analysis of the phosphoproteome.

**Offline fractionation via RP-HPLC at high pH**

Tryptic peptides were re-buffered in 20 mM ammonium formiate buffer pH 10, shortly before separation by reversed phase liquid chromatography at pH 10. The unbound fraction of the phosphopeptide enrichment was separated into 96 time-based fractions on a Phenomenex column (150 × 2.0 mm Gemini-NX 3 µm C18 110Å, Phenomenex, Torrance, CA, USA) using an Agilent 1200 series HPLC system fitted with a binary pump delivering solvent at 100 µL/min. Acidified fractions were consolidated into 36 fractions via a concatenated strategy described by Wang *et al.* (10). The bound fraction containing the phosphopeptides was separated into 20 fractions on a Dionex column (500 µm × 50 mm PepSwift RP, monolithic, Dionex Corporation, Sunnyvale, CA, USA) using an Agilent 1200 series nanopump delivering solvent at 4 µL/min. Peptides were separated by applying a gradient of 90% aceonitrile containing 20 mM ammonium formiate, pH 10 as described by (Gilar et al., 2005). After solvent removal in a vacuum concentrator, samples were reconstituted in 5% formic acid for LC-MS/MS analysis and kept at -80°C until analysis.

**2D-RP/RP liquid chromatography mass spectrometry**

Mass spectrometry was performed on an Orbitrap Fusion Lumos mass spectrometer (ThermoFisher Scientific, San Jose, CA) coupled to an Dionex Ultimate 3000RSLC nano system (ThermoFisher Scientific, San Jose, CA) via nanoflex source interface. Tryptic peptides were loaded onto a trap column (Pepmap 100 5μm, 5 × 0.3 mm, ThermoFisher Scientific, San Jose, CA) at a flow rate of 10 μL/min using 2% ACN and 0.1% TFA as loading buffer. After loading, the trap column was switched in-line with a 50 cm, 75 µm inner diameter analytical column (packed in-house with ReproSil-Pur 120 C18-AQ, 3 μm, Dr. Maisch, Ammerbuch-Entringen, Germany). Mobile-phase A consisted of 0.4% formic acid in water and mobile-phase B of 0.4% formic acid in a mix of 90% acetonitrile and 10% water. The flow rate was set to 230 nL/min and a 90 min gradient used (6 to 30% solvent B within 81 min, 30 to 65% solvent B within 8 min and, 65 to 100% solvent B within 1 min, 100% solvent B for 6 min before equilibrating at 6% solvent B for 18 min). Analysis was performed in a data-dependent acquisition mode. Full MS scans were acquired with a scan range of 375 - 1650 m/z in the orbitrap at a resolution of 120,000 (at 200 m/z). Automatic gain control (AGC) was set to a target of 2 × 10^5^ and a maximum injection time of 50 ms. Precursor ions for MS^2^ analysis were selected using a TopN dependant scan approach with a max cycle time of 3 seconds. MS^2^ spectra were acquired in the Orbitrap (FT) at a resolution of 50,000 (at 200 m/z). Precursor isolation in the quadrupole was set to 0.4 Da and 1.2 Da for the proteome and phosphoproteome, respectively. Higher energy collision induced dissociation (HCD) was used with a normalized collision energy (NCE) of 38%. AGC was set to 1 × 10^5^  with a maximum injection time of 105 ms and 118 ms for the proteome and phosphoproteome, respectively. Dynamic exclusion for selected ions was 60 s for the proteome and 20 s for the phosphoproteome. A single lock mass at *m/z* 445.120024 for recalibration was employed (11). Xcalibur version 4.0.0 and Tune 2.1 were used to operate the instrument.

**Phosphoproteomic analysis**

Acquired raw data files were processed using the Proteome Discoverer 2.2.0. platform, utilizing the Sequest HT database search engine and Percolator validation software node (V3.04) to remove false positives with a false discovery rate (FDR) of 1% on PSM and protein level under strict conditions. Searches were performed with full tryptic digestion against the mouse SwissProt database v2017.12 (25293 sequences and appended known contaminants) with up to two miscleavage sites. Oxidation (+15.9949 Da) of methionine, deamidation (+0.984016 Da) of asparagine and glutamine were set as variable modifications, whilst carbamidomethylation (+57.0214 Da) of cysteine residues and TMT 6-plex labelling of peptide N-termini and lysine residues were set as fixed modifications. For phosphopeptides phosphorylation (+79.9663 Da) of serine, threonine and tyrosine was additionally included as a variable modification. Data was searched with mass tolerances of ±10 ppm and 0.025 Da on the precursor and fragment ions (HCD), respectively. Results were filtered to include peptide spectrum matches (PSMs) with Sequest HT cross-correlation factor (Xcorr) scores of ≥1 and 1% FDR peptide confidence. The ptmRS algorithm was additionally used to validate phospopeptides with a set score cutoff of 75. PSMs with precursor isolation interference values of ≥ 50% or average TMT-reporter ion signal-to-noise values (S/N) ≤ 10 were excluded from quantitation. Isotopic impurity correction and TMT channel-normalization based on total peptide amount were applied. TMT channel assignment was as follows: 126: Calreticulin WT DMSO rep1, 127N: Calreticulin WT DMSO rep2, 127C: Calreticulin WT HEM treated rep1, 128N: Calreticulin WT HEM treated rep2, 128C: Calreticulin mutant DMSO re1, 129N: Calreticulin mutant DMSO rep2, 129C: Calreticulin mutant DMSO rep3, 130N: Calreticulin mutant HEM rep1, 130C: Calreticulin mutant HEM rep2, 131: Calreticulin mutant HEM rep3. Reporter channel abundances were normalized to equal total peptide signal in each TMT channel. The phosphoproteomics experiment was acquired in two technical replicates. Phosphopeptides observed only in a single technical replicate or those where the difference between the two technical measurements of any TMT channel was more than 20% of the most intense TMT channel of that phosphopeptide (averaged over the two replicates) were excluded from the further analysis. The arithmetic average of relative intensities (relative against the sum of all 10 reporter channels of the given technical replicate) were used as the final measure of the phosphopeptide abundance changes. As the last normalization step, the phosphopeptide abundances were further normalized for protein abundance changes. LIMMA statistical model (12) and Benjamini-Hochberg procedure (FDR correction) were used to calculate statistical significance of observed changes on protein and phosphopeptide level. TMT ratios with Q-values lower than 0.01 were considered as significant. The ratio thresholds used in Figures 5 are ±log_2_(1.45) and ±log_2_(1.15) for phosphoproteomics and proteomics respectively. These thresholds corresponded to the 5% quantile of the distribution of ratios observed among biological replicates within the same sample types (e.g. meaning that biologically replicated measurements of protein abundances within the same condition differ from each other by more than ±log_2_(1.15) only for 5% of quantified proteins).

Supplementary table

Supplementary table 1 List of antibodies used for western blot

| **Antibody** | **Species** | **Manufacturer** | **Catalog number** |
| --- | --- | --- | --- |
| **Primary Antibody** | | | |
| pRPA (S4/S8) | Rabbit | Bethyl Laboratories | A300-245A |
| pRPA (S33) | Rabbit | Bethyl Laboratories | A300-246A |
| RPA | Mouse | Abcam | ab2175 |
| HSC70 | Mouse | Santa Cruz Biotechnology | sc-7298 |
| yH2AX-S139 | Rabbit | Cell Signalling Technology | 9718 |
| pChk1-S345 | Rabbit | Cell Signalling Technology | 2348 |
| Chk1 | Mouse | Santa Cruz Biotechnology | sc-56291 |
| Mutant CALR specific antibody | - | Myelopro diagnostics and research |  |
| Calregulin (calreticulin) | Mouse | Santa Cruz Biotechnology | sc-373863 |
| c-MPL | Rabbit | Merck/Millipore | 06-944 |
| GAPDH | Mouse | Santa Cruz Biotechnology | sc-32233 |
| **Secondary Antibody** | | | |
| Rabbit IgG HRP linked Ab | - | Cytiva | GENA934 |
| Mouse IgG HRP linked Ab | - | Cytiva | GENA931 |

Supplementary table 2 List of antibodies used for immunofluorescence

| **Antibody** | **Species** | **Manufacturer** | **Catalog number** |
| --- | --- | --- | --- |
| **Primary Antibody** | | | |
| pRPA (S4/S8) | Rabbit | Sigma | PLA0071 |
| yH2AX-S139 | Mouse | Merck Millipore | JBW301 |
| Rabbit IgG Isotype Control | Rabbit | Invitrogen | 02-6102 |
| Mouse IgG1 kappa Isotype control | Mouse | eBioscience | 14-4714-82 |
| **Secondary Antibody** | | | |
| Anti-rabbit IgG (H+L) Alexa Fluor 488 | Goat | Invitrogen | A-11034 |
| Anti-mouse IgG (H+L) Alexa Fluor 647 | Goat | Invitrogen | A-21235 |

**Supplementary figure**


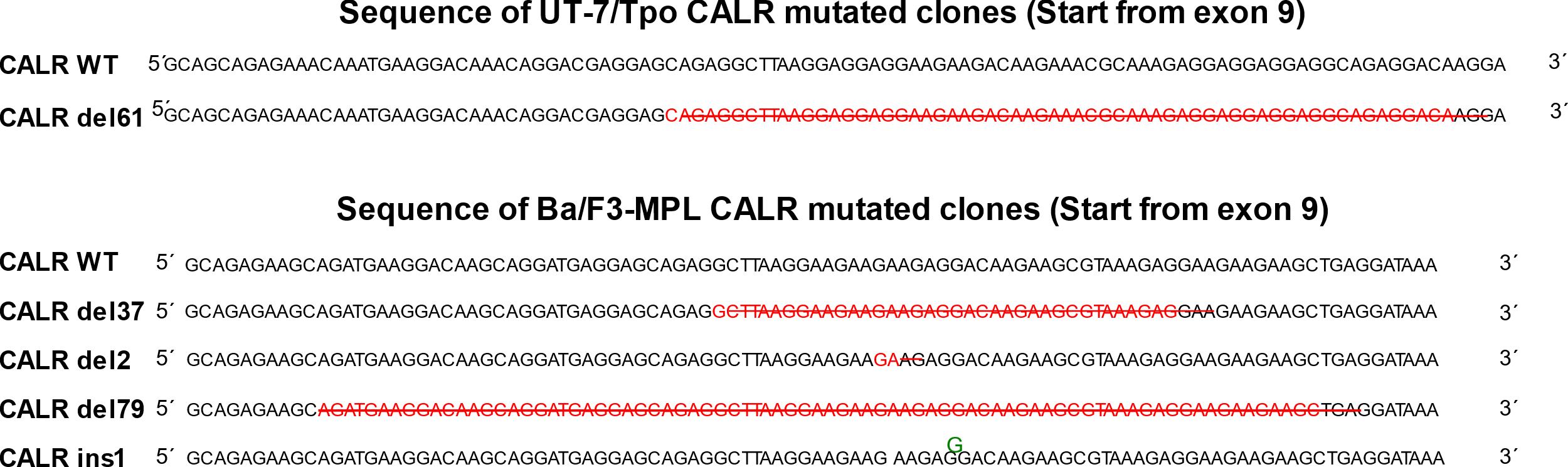


**Supplementary figure 1 Sequence information of CRISPR-Cas9 generated CALR mutated clones**

DNA sequences at CALR exon 9 of CALR mutated clones used in the study are shown. Deletion is annotated as red color and strikethrough. Insertion is shown in green color.


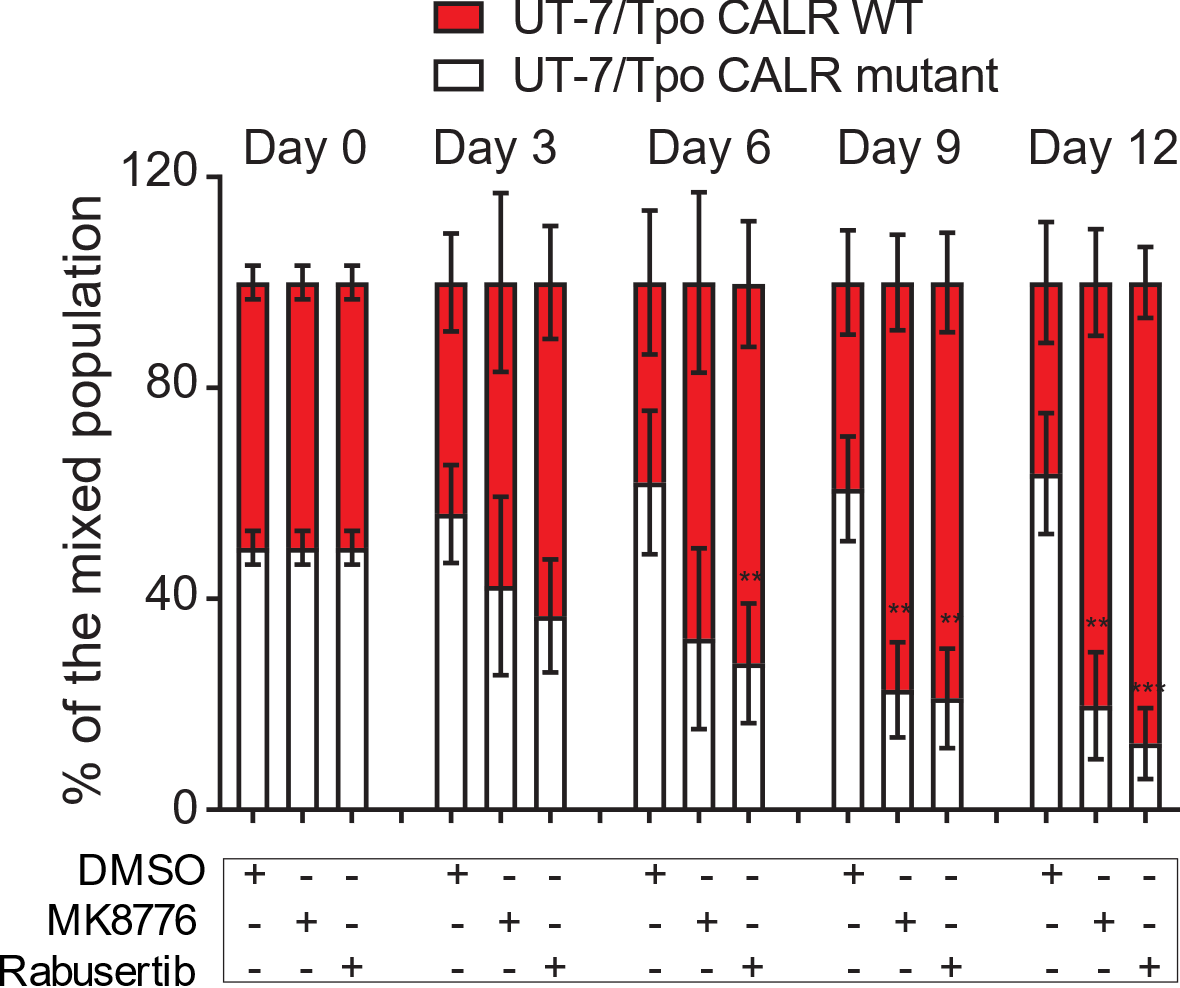


**Supplementary figure 2 Co-culture competition assay in UT-7/Tpo cell lines with the reverse-color setup.**

UT-7/Tpo *CALR* wild type (mCherry positive) and del61/WT cell line (mCherry negative) were mixed at 1:1 ratio and were incubated with 0.8 µM of CHK1 inhibitor Rabusertib or 1 µM of MK8776 over 12 days.


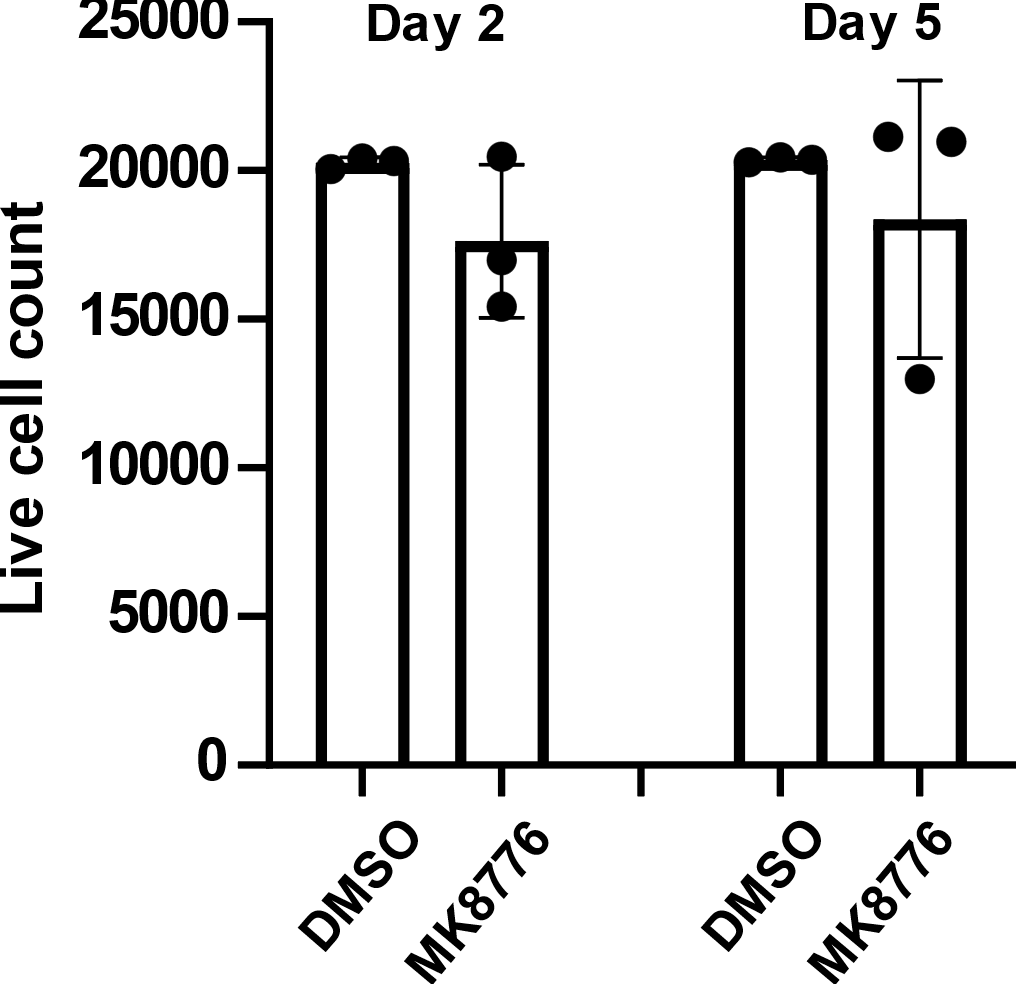


**Supplementary figure 3 Absolute live cell count of Ba/F3-MPL cells after MK8776 treatment.**

BaF3-MPL CALR wild type and del37/del37 cell line were incubated with 1 µM MK8776 over 5 days. After cell suspension was collected at day 2 and day 5 time points, the same volume of cell suspension of each sample was recorded in the FACs readout to count the absolute cell number. The recorded live cell number was plotted.


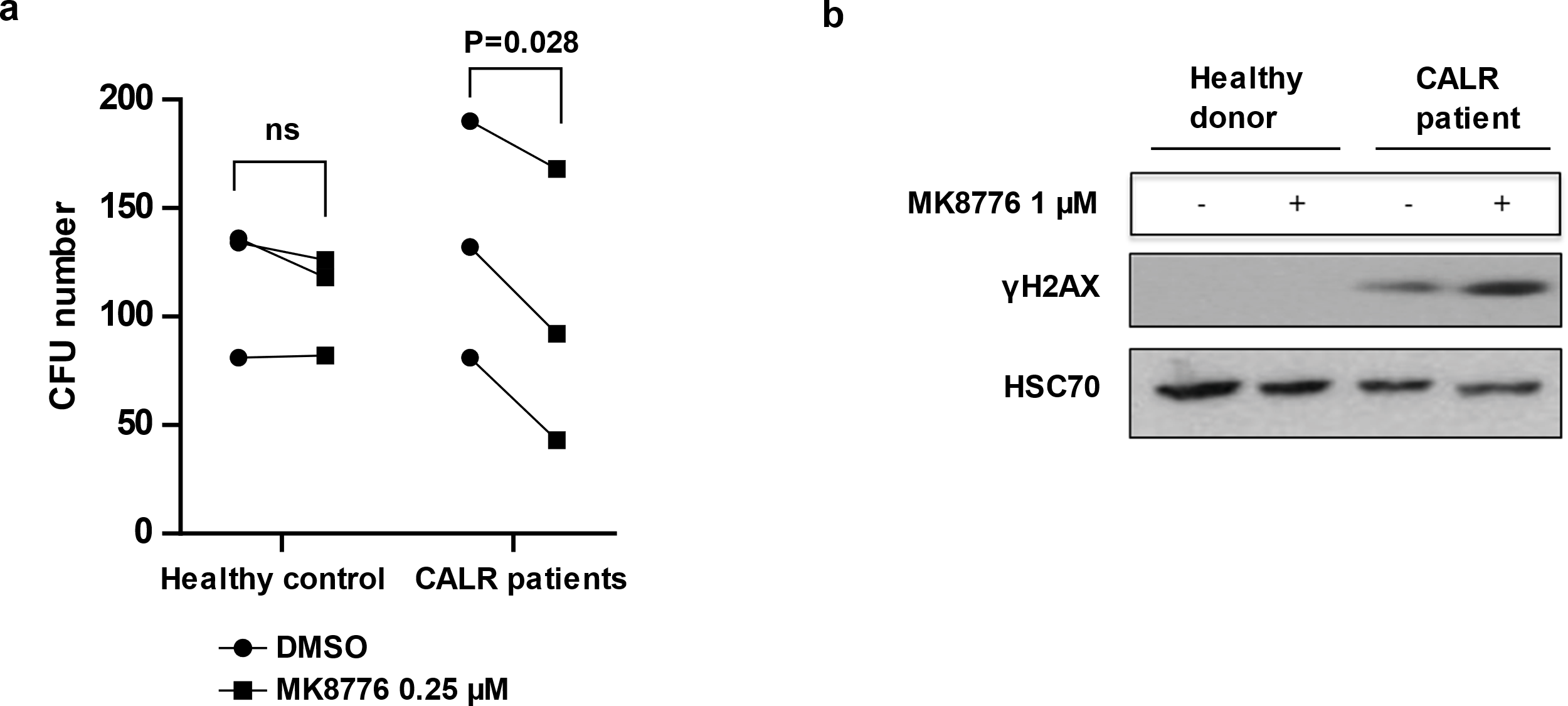


**Supplementary figure 4 Colony formation assay of healthy individuals and CALR patients following MK8776 treatment.**

CD34+ cells were isolated from PBMCs of 3 healthy individuals and 3 CALR patients. Colonies number was counted 10 days after 0.25 µM of MK8776/DMSO treatment.


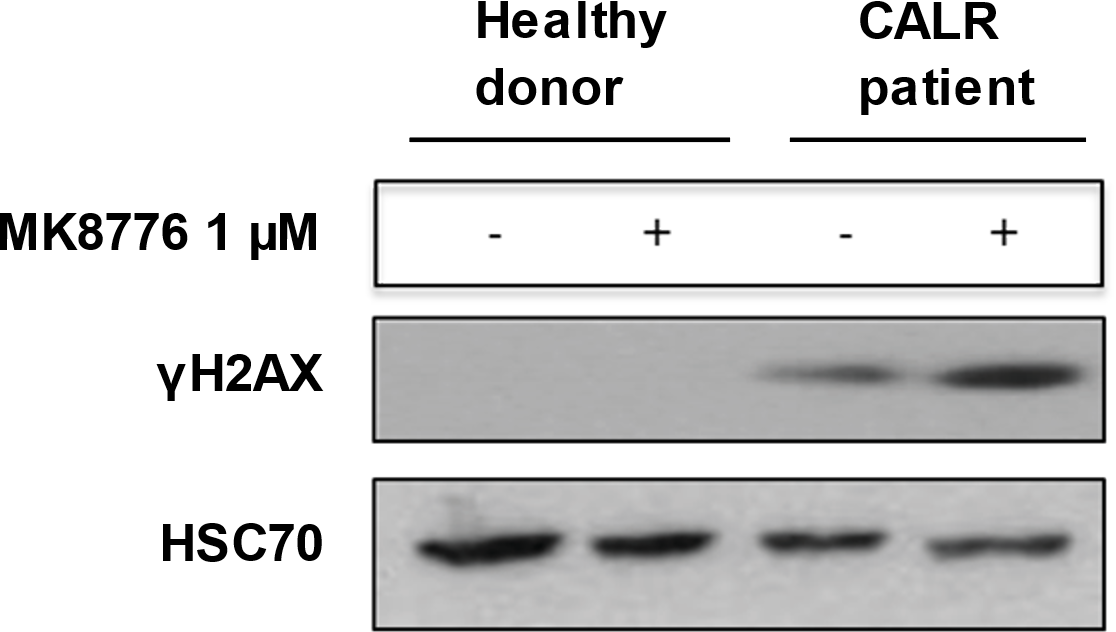


**Supplementary figure 5 Western blot analysis of the CD34+ cells from 1 healthy individual and 1 CALR ins5 patient following 1µM MK8776 treatment.**


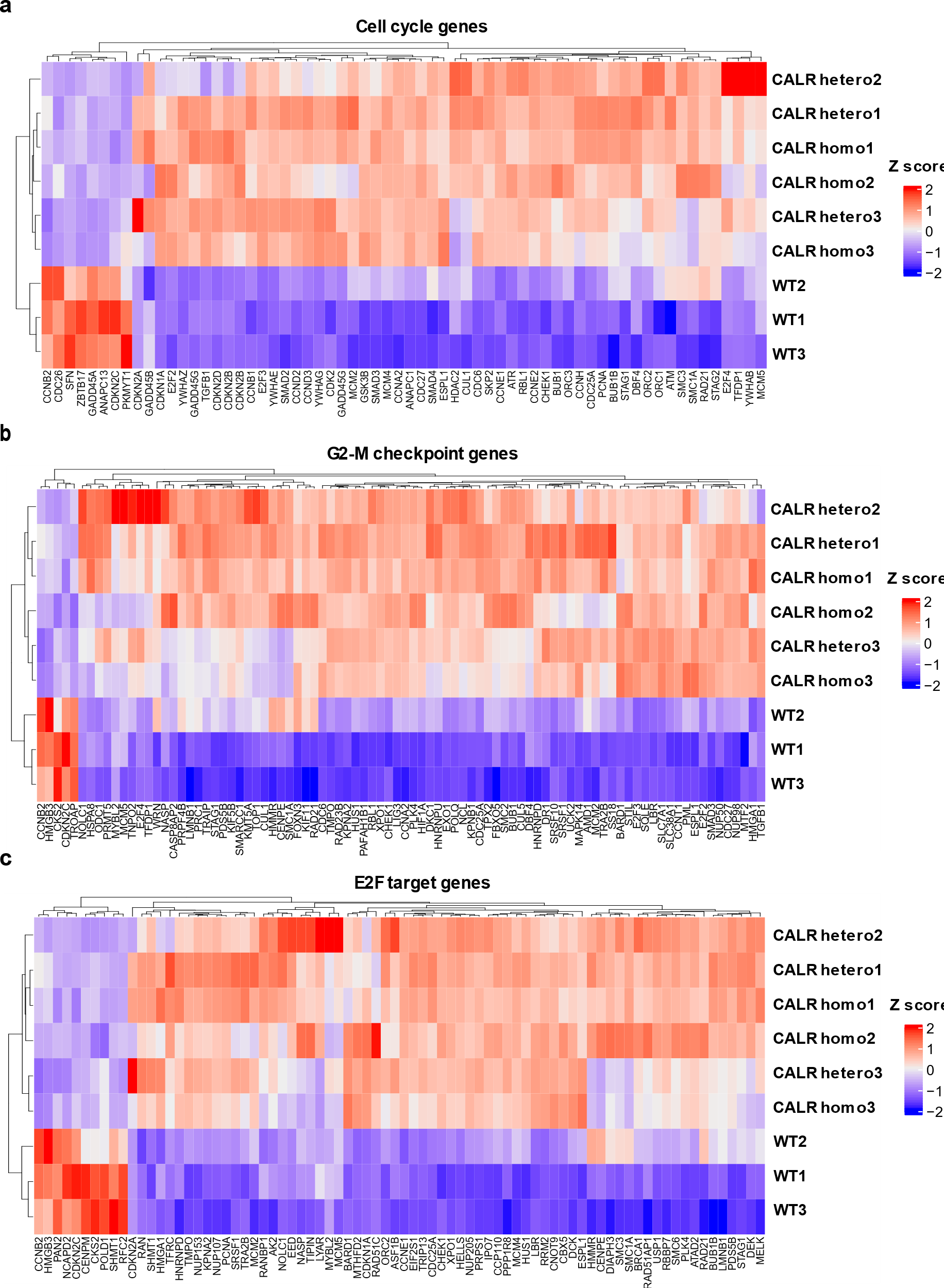


**Supplementary figure 6 Heatmap of differentially expressed cell cycle related genes**

The differentially expressed genes in three cell cycle related gene sets (a) Cell cycle (b) G2-M checkpoint (c) E2F target were plotted in heatmaps. The Z-score and the plotting of heatmaps were performed using R package ComplexHeatmap (5). A higher z-score indicates a higher relative expression level.

**Supplementary references**

1. Broad Institute. Picard Toolkit.. GitHub Repository. 2019. Available from: http://broadinstitute.github.io/picard/

2. Dobin A, et al. STAR: ultrafast universal RNA-seq aligner. Bioinformatics. 2013 Jan;29(1):15–21.

3. Love MI, Huber W, Anders S. Moderated estimation of fold change and dispersion for RNA-seq data with DESeq2. Genome Biol. 2014 Dec 5;15(12):550.

4. Wickham H. ggplot2: Elegant Graphics for Data Analysis. Springer-Verlag New York; 2016. Available from: https://ggplot2.tidyverse.org.

5. Gu Z, Eils R, Schlesner M. Complex heatmaps reveal patterns and correlations in multidimensional genomic data. Bioinformatics. 2016 Sep 15;32(18):2847–9.

6. Chen EY, et al. Enrichr: interactive and collaborative HTML5 gene list enrichment analysis tool. BMC Bioinformatics. 2013;14(1):128.

7. Kuleshov M V., et al. Enrichr: a comprehensive gene set enrichment analysis web server 2016 update. Nucleic Acids Res. 2016 Jul 8;44(W1):W90–7.

8. Xie Z, et al. Gene Set Knowledge Discovery with Enrichr. Curr Protoc. 2021 Mar 29;1(3):1–51.

9. Wiśniewski JR, Zougman A, Nagaraj N, Mann M. Universal sample preparation method for proteome analysis. Nat Methods. 2009;6(5):359–62.

10. Wang Y, et al. Reversed-phase chromatography with multiple fraction concatenation strategy for proteome profiling of human MCF10A cells. Proteomics. 2011 May;11(10):2019–26.

11. Olsen J V., et al. Parts per Million Mass Accuracy on an Orbitrap Mass Spectrometer via Lock Mass Injection into a C-trap. Mol Cell Proteomics. 2005 Dec;4(12):2010–21.

12. Smyth GK. Linear Models and Empirical Bayes Methods for Assessing Differential Expression in Microarray Experiments. Stat Appl Genet Mol Biol. 2004 Jan 12;3(1):1–25.
